# Antigen-specific TIL therapy for melanoma: a flexible platform for personalized cancer immunotherapy

**DOI:** 10.1186/2051-1426-1-S1-P19

**Published:** 2013-11-07

**Authors:** Sander Kelderman, Bianca Heemskerk, Mireille Toebes, Marit van Buuren, Nienke van Rooij, Laura Bies, Lorenzo Fanchi, Lothar Germeroth, Pia Kvistborg, Ton Schumacher

**Affiliations:** 1 Netherlands Cancer Institute NKI-AVL, Amsterdam, the Netherlands; 2 Stage Cell Therapeutics, Göttingen, Germany

## 

Adoptive cell therapy (ACT) for melanoma has shown to be an effective treatment modality with clinical responses in approximately 50% of stage IV disease patients. However, durable complete responses are observed in only a small proportion (10-20%) of patients. Recent data have demonstrated that the tumor-infiltrating lymphocyte (TIL) infusion products used for therapy harbor only low frequencies of T cells reactive against presently known shared melanoma-associated epitopes. Furthermore, ongoing studies indicate that T cell reactivity against patient-specific mutated antigens may be common in human melanoma. Collectively, these two observations suggest that the development of a clinically applicable approach that allows the selection of defined tumor-reactive T cell populations in a patient-specific manner would be of value. Here we develop such a platform technology and demonstrate its value in a preclinical model. First, reversible (strep-tagged) HLA-A*0201 molecules for clinical use were produced with a UV-sensitive peptide ligand, and the resulting MHC class I monomers could be loaded with peptides of interest in straightforward peptide exchange reactions. To subsequently evaluate the potential of these MHC exchange streptamers for antigen-specific T cell enrichment, we performed individual or combined enrichments of melanoma-reactive T cell populations from TIL populations. Enrichment of both high (1≥%) and low frequency (<1%) tumor-specific T cell populations, either alone or combined, was shown to be feasible with a mean enrichment factor of 27 and 56. To evaluate the potential of this technology for the generation of T cell products that recognize patient-specific neo-antigens, we performed combined enrichments of two HLA-A*0201 restricted T cell responses, directed against neo-antigens in the CDK4 (2.2% of CD8+ cells) and GCN1L1 (0.58% of CD8+ cells) gene products that we identified by exome sequencing. Combined enrichment of these two patient-specific T cell populations resulted in a neo-antigen specific cell product with a purity of 92.4%. Functional analysis of the neo-antigen selected T cell product against autologous tumor in NSG mice showed superior tumor control and survival compared to non-selected ‘standard’ TIL. Together, these data demonstrate that selection of (panels of) antigen-specific T cells using the combination of UV-induced ligand exchange and reversible streptamer technology is feasible. In combination with exome-driven analysis of tumor-specific T cell reactivity in human cancer, this strategy forms a highly flexible platform for the development of antigen-specific cell products for personalized cancer immunotherapy.

